# In Vitro and In Vivo Evaluation of Alectinib-Loaded Dendrimer Nanoparticles as a Drug Delivery System for Non-Small Cell Lung Carcinoma

**DOI:** 10.3390/pharmaceutics17080974

**Published:** 2025-07-28

**Authors:** Mahmood R. Atta, Israa Al-Ani, Ibrahim Aldeeb, Khaldun M. AlAzzam, Tha’er Ata, Mohammad A. Almullah, Enas Daoud, Feras Al-Hajji

**Affiliations:** 1 Department of Pharmaceutics and Pharmaceutical Technology, Faculty of Pharmacy, Pharmacological and Diagnostic Research Center (PDRC), Al-Ahliyya Amman University, Amman 19328, Jordan; thaerata7@gmail.com (T.A.); mohammadaref988@gmail.com (M.A.A.); esolayman@ammanu.edu.jo (E.D.); 2 Faculty of Pharmacy, Zarqa University, Zarqa 13132, Jordan; ialdeeb@zu.edu.jo; 3 Department of Chemistry, Faculty of Science, The University of Jordan, Amman 11942, Jordan; k.azzam@ju.edu.jo; 4 Faculty of Pharmacy, Applied Science University, Amman 11937, Jordan; f_elhajji@asu.edu.jo

**Keywords:** in vivo, in vitro, Alectinib, polyamidoamine dendrimer, xenograft, pharmacokinetics, non-small-cell lung cancer

## Abstract

**Background/Objectives**: Alectinib, a second-generation tyrosine kinase inhibitor indicated for the treatment of non-small-cell lung cancer (NSCLC), exhibits suboptimal oral bioavailability, primarily attributable to its inherently low aqueous solubility and limited dissolution kinetics. This study aimed to enhance Alectinib’s solubility and therapeutic efficacy by formulating a G4-NH2-PAMAM dendrimer complex. **Methods**: The complex was prepared using the organic solvent evaporation method and characterized by DSC, FTIR, dynamic light scattering (DLS), and zeta potential measurements. A validated high-performance liquid chromatography (HPLC) method quantified the Alectinib. In vitro drug release studies compared free Alectinib with the G4-NH2-PAMAM dendrimer complex. Cytotoxicity against NSCLC cell line A549 was assessed using MTT assays, clonogenic assay, and scratch-wound assay. Xenograft effect was investigated in the H460 lung cell line. Pharmacokinetic parameters were evaluated in rats using LC–MS/MS. **Results**: Alectinib exhibited an encapsulation efficiency of 59 ± 5%. In vitro release studies demonstrated sustained drug release at pH 6.8 and faster degradation at pH 2.5. Anticancer activity in vitro showed comparable efficacy to free Alectinib, with 98% migration inhibition. In vivo tumor suppression studies revealed near-complete tumor regression (~100%) after 17 days of treatment, compared to 75% with free Alectinib. Pharmacokinetic analysis indicated enhanced absorption (shorter Tmax), prolonged systemic circulation (longer half-life), and higher bioavailability (increased AUC) for the dendrimer-complexed drug. **Conclusions**: These findings suggest that the G4-NH2-PAMAM dendrimer system significantly improves Alectinib’s pharmacokinetics and therapeutic potential, making it a promising approach for NSCLC treatment.

## 1. Introduction

Cancer refers to a heterogenous group of more than one hundred diseases that evolve progressively over time. The Oxford Concise Medical Dictionary defines cancers as malignant neoplasms—both carcinoma and sarcoma— arising from abnormal, uncontrolled proliferation of cells that subsequently invade and destroy surrounding tissues. Although individual cancers differ widely in their clinical presentation, the core molecular pathways that drive tumor initiation and progression are remarkably conserved [1].

Historically, lung cancer has been divided into small-cell lung cancer (SCLC) and non-small-cell lung cancer (NSCLC). Advances in molecular pathology have refined this framework, revealing discrete genetic biomarkers and signaling aberrations that underpin each subtype [2]. NSCLC accounts for approximately 85% of all lung cancer cases and remains a leading cause of cancer-related mortality worldwide. Originated in bronchial and alveolar epithelial cells, NSCLC commonly metastasizes to regional and distant organs. Histopathologically, it is further classified into adenocarcinoma, squamous cell carcinoma, and large-cell carcinoma [3].

In the United States, lung cancer is the second most frequently diagnosed malignancy yet the foremost cause of cancer deaths. In 2020 alone, an estimated 247,270 new cases of lung cancer were reported. Between 2010 and 2016, the overall five-year relative survival rate for NSCLC in the US was 26.5% [4]. Cigarette smoking remains the primary risk factor for lung cancer, with cancer statistics of 2022 indicating that more than 80% of lung cancer patients had a history of smoking. Compared to non-smokers, individuals with a history of smoking have an approximately 4- to 10-fold increased risk of developing lung cancer [5].

Targeted therapy with small-molecule tyrosine kinase inhibitors (TKIs) has emerged as a promising therapeutic approach for lung cancer, offering tumor regression with reduced toxicity. In patients whose tumors harbor actionable driver mutations, TKIs have extended median overall survival from <10 months to nearly 40 months while reducing systemic toxicity [6].

Alectinib (9-ethyl-6,6-dimethyl-8-[4-(morpholine-4-yl) piperidin-1-yl]-11-oxo-6,11-dihydro-5H-benzo[b]carbazole-3-carbonitrile hydrochloride) is a highly selective anaplastic lymphoma kinase (ALK) inhibitor with excellent central nervous system (CNS) penetration. It effectively suppresses resistance mutations such as Leu1196Met and Cys1156Tyr, demonstrating significant in vitro and in vivo efficacy in ALK-positive NSCLC, particularly in chemotherapy-pretreated but ALK-TKI-naïve patients [7]. As a first-line treatment, Alectinib has shown greater efficacy and lower toxicity than Crizotinib, with disease progression or mortality observed in 68% of Crizotinib-treated patients versus 41% of those receiving Alectinib [8].

Despite its clinical value, Alectinib’s formulation is hampered by poor water solubility and limited bioavailability. The hydrophobic benzo–carbazole scaffold yields a permeability of 2.5 × 10^−4^ cm/s and an absolute bioavailability of merely 37% [9]. Similar solubility–permeability barriers affect nearly 40% of new drug candidates, prompting growing interest in nanocarrier-based delivery strategies.

Dendrimers are particularly attractive nanocarriers owing to their highly branched, monodisperse architecture, tunable size, and capacity to enhance drug solubility [10]. Dendrimers are synthetic macromolecules with a three-dimensional globular structure, monodispersity, and nanoscale dimensions. The first extensively studied dendritic structures were poly(amidoamine) (PAMAM) dendrimers, synthesized using a divergent growth approach with ammonia or ethylenediamine as initiator cores [11]. PAMAM dendrimers offer a large surface area capable of accommodating significant drug payloads, making them suitable for drug delivery applications [12].

Given that Alectinib is classified in Biopharmaceutical Classification System (BCS) Class IV, improving its aqueous solubility and dissolution rate is essential for maximizing therapeutic benefit. PAMAM dendrimers offer a promising nanodrug delivery approach, enabling higher drug loading, controlled release, and improved pharmacokinetics. However, the use of G4-NH_2_ PAMAM dendrimers for Alectinib delivery remains unexplored. The present study investigates G4-NH_2_ PAMAM dendrimers as a novel nanocarrier to optimize the pharmacokinetic and pharmacodynamic profile of Alectinib, enhancing its therapeutic efficacy in NSCLC.

## 2. Materials and Methods

### 2.1. Material

A wide range of chemicals and materials were utilized in this study, each playing a vital role in conducting the experimental procedures. Alectinib hydrochloride (>99.9%) was purchased from Biosynth Zurich (Switzerland, distributed by Cymit Química S.L., Barcelona, Spain), polyamidoamine dendrimer generation 4 (PAMAM dendrimers G4, 1 g vial, molecular weight = 14,215 g/mol, 64 amine end groups) was purchased from Dendritech, Inc., Midland, MI, USA, dimethyl sulfoxide (DMSO) (99.99%) from EMSURE^®^, Charlotte, NC, USA, methanol (HPLC grade) (99.99%) from Honeywell International Charlotte, NC USA, 3-(4,5-dimethyldiazol-2-yl)-2,5-diphenyltetrazolium bromide (MTT) Cell Proliferation Kit I was purchased from Sigma-Aldrich (St. Louis, MO, USA), polyvinyl alcohol (PVA), Tween 80, PEG400, A549 lung cancer cells, and H460 large lung cancer cells were purchased from American Type Culture Collection (ATCC), phosphate-buffered saline from Sigma Aldrich, St. Louis, MO, USA, crystal violet from Merck Millipore (Burlington, MA, USA), Medium Roswell Park Memorial Institute (RPMI 1640) was purchased from Thermo Fisher Scientific, Waltham, MA, USA, trypsin—EDTA was from Euro Clone in Pero, Italy, and ^®^Matrigel was from Corning (New York, NY, USA).

### 2.2. UV-VIS Scan for λ Max of Alectinib and G4-NH2-PAMAM Dendrimer

To analyze and quantify free Alectinib and G4-NH2-PAMAM dendrimer, a UV spectrophotometer was selected. A stock solution was prepared by dissolving 5 mg of Alectinib in 25 mL of methanol, followed by filtration to obtain a clear solution.

The samples were then filtered and scanned using a UV spectrophotometer (model U-2000, Hitachi, Tokyo, Japan) equipped with a quartz cuvette, using methanol as the blank. The absorbance spectra were recorded over a wavelength range of 200–400 nm to identify characteristic absorption peaks. This method ensured the reliability and accuracy of detecting both Alectinib and PAMAM dendrimer components.

### 2.3. Instrumentation and Chromatographic Conditions

The HPLC method with photodiode array (PDA) detection was used for the estimation of Alectinib. The method was developed and validated on Hipersil C18- (150 mm × 4.6 mm, 5 µm). The HPLC system utilized the Millennium software, version 32, for data processing and analysis. The eluent/solvent system was composed of methanol:acetonitrile:water at a ratio of 40%:30%:30% *v*/*v*. The solvent system flowed with a flow rate of 1.0 mL/min. The detection of Alectinib was carried out at 340 nm based on UV scanning of the compound and reported data in the literature [13,14]. The samples (20 µL) were introduced into the system via a Waters autosampler. The suggested method was partially validated for linearity, accuracy, precision, specificity, and selectivity. The mobile phase was prepared by taking 500 mL of HPLC grade water and adding 1 drop of orthophosphoric acid 85% to adjust the pH to 3.8, then 300 mL of acetonitrile and 400 mL of methanol were added to the previously prepared 500 mL of solution, mixed well, filtered through a 0.22 µm nylon membrane, and sonicated for 10 min for degassing prior to usage.

### 2.4. Preparation of Alectinib–Dendrimer Nanoparticles Using G4-NH2-PAMAM Dendrimers

The initial experiment to load Alectinib onto G4-PAMAM was conducted as described by Fatani et al. [15]:

First, 10 mg of Alectinib was dissolved in 5 mL of dimethyl sulfoxide (DMSO) to obtain a 2% *w*/*v* solution. Subsequently, 20 µL of G4-PAMAM dendrimer solution (containing 2 mg of dendrimers) was added to the drug solution and stirred at 500 rpm on a magnetic stirrer for 24 h. Following this, 10 mL of deionized water was added and the mixture was stirred under the same conditions for an additional 24 h at room temperature, resulting in the final dispersion of 15 mL.

To remove DMSO, the mixture was then stirred continuously at an elevated temperature of 40 °C for another 24 h. Due to the photosensitive nature of Alectinib, all procedures were performed under light-protective conditions using aluminum foil to cover the equipment. The resulting dispersion was subsequently evaluated for Alectinib loading efficiency.

### 2.5. Optimization of the Formulation After Synthesis of Alectinib–Dendrimer Nanoparticles

In this study, multiple formulations were developed to optimize the most effective composition. Various additives were incorporated into the basic formula as shown in Table 1. A fixed amount of Alectinib (10 mg) was used across all preparations, combined with different surfactants and stabilizers. The required quantities of these additives were accurately calculated and added to the reaction mixture, which were then brought to the final volume of 15 mL with deionized water. Each mixture was stirred in the dark at 25 °C for 24 h.

A total of nine formulations were prepared: F1 is the basic formula mentioned above, F2 had a higher ratio of PAMAM, F3, F4, and F5 used three concentrations of PVA as both a surfactant and stabilizer. F6 used PEG400 and F7 used Tween 80. The purpose of the addition of surfactants was to break the interfacial tension between Alectinib and dendrimers in an aqueous solution to facilitate its loading. F8 used a mixture of PVA and Tween 80 and F9 used a higher ratio of PAMAM to Alectinib (2.5:1) with 10 mg PVA. The formulations were prepared sequentially based on the encapsulation efficiency results obtained from previous batches.

Additives such as PVA and Tween 80 were dissolved in DMSO together with Alectinib and the formulations were prepared following the established procedure.

The resulted NPs were purified by filtration to remove the excess unincorporated drug and eliminate precipitated Alectinib. The filtrates were then lyophilized for 72 h using a Labcono Free Zone 6 Liter Benchtop Freeze Dry System and stored at −70 °C for further use.

### 2.6. Characterization of Alectinib–Dendrimer Nanoparticles by Measurement of Particle Size Distribution, PDI, and Zeta Potential

Following the preparation of each formula, a freeze dryer was utilized to gather the complexes in powdered form for characterization. The PS and PDI and zeta potential of Alectinib complexes and PAMAM dendrimers were measured using a Zeta sizer instrument (Malvern Instruments Ltd., Malvern, UK) that utilizes dynamic light scattering (DLS) [16]. The particle size was measured at neutral pH (water). For the measurement, 1 mL of each formulation was diluted 100 times with water and subjected to particle size measurement. The DLS analysis was carried out three times at a temperature of 25 °C and a scattering angle of 90°. The volume-average PS was calculated for each sample and average ± SD was recorded in a graph [17].

### 2.7. Entrapment Efficiency and Drug Loading

With approximately 0.010 g of Alectinib in each dendrimer PAMAM nanoparticle, the EE of the drug into the dendritic structure of G4-NH2-PAMAM dendrimers was studied to estimate the maximum drug encapsulation efficiency of each complex. To achieve this, the EE% of G4 complexes was determined by using the centrifugation method. The complexes were placed into a centrifuge tube and centrifugated at a speed of 2000 rpm for 5 min. Then, a sample from the supernatant was withdrawn and measured by an HPLC-UV spectrophotometer at 340 nm to determine the amount of free drug that was not entrapped in the nanoformulation. The following equations were used to measure the PAMAM dendrimer EE% [18].

(1) $$EE\%=\frac{Amount\;of\;total\;drug-Amount\;of\;free\;drug*100}{Amount\;of\;total\;drug}$$

While loading efficiency (LE) was measured using the following equation [19]:

$$LE\;\%=\frac{Amount\;of\;encapsulated\;drug}{Amount\;of\;encapsulated\;drug+amount\;of\;carrier\;used}*100$$

### 2.8. Fourier Transform Infrared (FTIR) Spectroscopy

The FTIR instrument utilized for scanning was the Shimadzu Irprestige 21 from Japan. The scanning range was set from 400 to 4000 cm^−1^. A minute amount of Alectinib, PAMAM, and the selected complex was combined with KBr and compressed into small discs, which were subsequently inserted into the apparatus. The prominent peaks in the spectra were analyzed and compared following the data that has been previously reported [19].

### 2.9. Differential Scanning Calorimetry (DSC)

DSC was performed on selected samples for further confirmation of the physical interaction between Alectinib and the PAMAM dendrimer. DSC (Model DT-60, Shimadzu, Kyoto, Japan) was used to perform this test. Alectinib powder and the G4-NH2-PAMAM–Alectinib complex were scanned from 0–380 °C at a rate of 10 °C/min. Thermograms were recorded and examined.

### 2.10. In Vitro Drug Release Studies Using a Dialysis Method in PBS (pH 6.8 and pH 2.5)

In vitro drug release studies were performed using the dialysis method [20]. A drug release study was conducted in vitro to measure the amount of drug released from the complex. Free Alectinib was used as a control. To carry out this investigation, two phosphate-buffered saline (PBS) solutions were prepared with different pH levels: one with a pH of 6.8 and another with a pH of 2.5. Based on a previous study, 1.5% SLS was added to the release media to keep the sink condition [13].

The dissolution study was conducted using the USP II apparatus (paddle) under the following conditions: a stirring speed of 100 rpm, a temperature of 37.0 ± 0.5 °C, and volume of release media of 200 mL. The NP samples were placed in a plastic tube sealed with a dialysis membrane with a molecular weight cut-off (MWCO) of 14,000 Da, which was previously soaked in either buffer for 24 h. A sample of the NPs, which is equivalent to 3.5 mg of Alectinib, was utilized for the release study. At predetermined time intervals, 1 mL aliquots were withdrawn and promptly replaced with fresh media. The collected samples were filtered by a 0.22 µm filter membrane and were subsequently injected into the HPLC system at a wavelength of 340 nm.

### 2.11. Cell Culturing and Cytotoxicity Assay

The A549 cell line, composed of human alveolar basal epithelial adenocarcinoma cells, was used for the in vitro evaluation. Cells were obtained from ATCC through the Faculty of Pharmacy at Al-Ahliyya Amman University. To maintain the A549 lung cancer cell line, Roswell Park Memorial Institute (RPMI) was employed. The medium was supplemented with 2 mL of L-glutamine, 50 mL of fetal bovine serum (FBS), and 5 mL of penicillin–streptomycin. The frozen A549 cell vials, stored in liquid nitrogen at approximately −196 °C, were quickly thawed at 37 °C in a water bath. The contents were then diluted with 10 mL of pre-warmed media and then centrifuged at 1500 rpm for 10 min. After discarding the supernatant, the tube was gently agitated to disperse the cells, and fresh medium (15 mL) was added. The cells were transferred to a T-25 flask and incubated in a tissue culture incubator at 37 °C with 5% carbon dioxide (CO_2_). Their confluency level was maintained within the ideal range of 80–90%. To assess cell viability, a 1:1 mixture of 50 µL of Trypan blue stain and 50 µL of cell solution was prepared in an Eppendorf tube. Then, 5 × 10^3^ cells were inoculated into individual wells of 96-well microplates using 100 μL of medium. The wells were then incubated overnight at 37 °C in a humidified incubator containing 5% CO_2_ to promote cell adhesion. After 24 h, a series of concentrations of G4-NH2-PAMAM–Alectinib NPs and free Alectinib were introduced into each well. The concentrations added to each well ranged from 100 μM/mL to 0.781 μM/mL. Negative control wells received 0.1% DMSO, while blank wells were filled with RPMI medium without cells. The plates were incubated for 72 h under standard conditions. Following the incubation period, 20 μL of MTT reagent was added to each well, and the plate was re-incubated at 37 °C for 4 h to facilitate the reduction of MTT and production of formazan crystals. Once the medium containing MTT was removed, 120 μL of DMSO was introduced into each well to dissolve the formazan crystals that had formed. The absorbance of the formazan solution in each well was promptly quantified at 590 nm utilizing a 96-well plate reader manufactured by Biotech, and the IC_50_ values were calculated according to Equation (2) [21,22].

(2) $$\mathrm{Cell}\;\mathrm{survival}\;\mathrm{rate}=\frac{\;(1-\left(\mathrm{ABS}-\mathrm{MIN}\right))}{\left(\mathrm{MAX}-\mathrm{MIN}\right)}\times 100\%$$

### 2.12. Clonogenic Assay

A 6-well plate was used to seed 600 cells into each well, along with 2 mL of culture medium. The plate was then placed in an incubator set at 37 °C in a humidified atmosphere containing 5% CO_2_ for 24 h to allow for cell attachment. After the initial incubation, the medium in each well was replaced with 2 mL of treatment or negative control. Triplicate wells were treated with concentrations equivalent to 0.5 IC_50_, IC_50_, and 2 IC_50_ of free Alectinib, while others were treated with 0.5 IC_50_, IC_50_, and 2 IC_50_ of G4-NH2-PAMAM–Alectinib complexes. The cells in the other untreated group served as the control. The plate was incubated at 37 °C with 5% CO_2_ for 3 days in the incubator. After the 3-day incubation period, the treatment or negative control was discarded and replaced with fresh media. At the end of this experiment (about 10 days), each well was washed with PBS exposed to 4% paraformaldehyde and re-incubated for 1 h. After that, 2% of crystal violet was added to each well and left for another 1 h [23]. Finally, the dye was washed and left overnight until dry. The colonies in each well were then counted manually and % of inhibition was calculated as follows:

(3) $$\%\;of\;inhibition=\frac{\left(No.\;of\;colonies\;of\;control-No.of\;colonies\;treated\right)}{No.\;of\;colonies\;of\;control}\times 100\%$$

### 2.13. Scratch Assay for Cell Migration

A549 cells were seeded into 6-well plates at a density of 2 × 10^5^ cells per well and incubated overnight at 37 °C in a humidified incubator with 5% CO_2_ using RPMI 1640 medium. After the cells reached full confluence and formed a uniform monolayer, a scratch approximately 1 mm wide was created across the cell layer using a sterile 200 μL micropipette tip. The wells were then gently washed with phosphate-buffered saline (PBS) to remove cellular debris.

Subsequently, triplicate wells were treated with G4-NH_2_-PAMAM–Alectinib NPs at concentrations equivalent to 0.5 IC_50_, IC_50_, and 2IC_50_ each dissolved in 2 mL of medium. Complete growth media containing media and cells for the complexes and 0.1% DMSO for free Alectinib were used to treat the negative control wells. To evaluate wound closure, images of the scratch area were captured at the start of the experiment (0 h) and after 72 h of treatment. Image analysis was performed using ImageJ^®^ software (version 1.4.3.67, Symmetry Software) to measure the wound area and assess cell migration quantitatively

### 2.14. In Vivo Xenograft Study

This study involved 24 male BALC mice, aged 5 weeks and weighing 20 ± 2 g, housed under controlled environmental conditions (temperature: 25.2 °C, humidity: 60  ±  5%, 12 h light–dark cycle) with ad libitum access to standard rodent chow and autoclaved water. All animal procedures were ethically approved by the IRB of Al-Ahliyya Amman University (APU: AAU 4/6/2022-2023) and conducted at the Pharmacological and Diagnostic Research Center (PDRC). Human lung large-cell carcinoma (H460) cells were cultured in RPMI 1640 medium, prepared through trypsinization and centrifugation, and underwent viability testing with Trypan blue exclusion, then were suspended in ECM Matrigel^®^. After 21 days of immunosuppression with 5 mg/kg dexamethasone administered intraperitoneally each mouse was subcutaneously injected in the right flank with 2.5 × 10^6^ viable H460 cells. Tumor growth was monitored for 14 days using caliper measurements. This was followed by a 17-day treatment study where mice received either 20 mg/kg G4-NH2-PAMAM–Alectinib NPs, 20 mg/kg Alectinib alone, or DW to evaluate the antitumor effects of the treatments.

### 2.15. Pharmacokinetic Study and Measurement of Bioavailability by Liquid Chromatography–Tandem Mass Spectrometry (LC-MS-MS) for Analysis of Alectinib in Rats’ Plasma

Quantification and identification of Alectinib were performed using liquid chromatography coupled with mass spectrometry (LC/MS), employing rosuvastatin as the internal standard (IS). The analysis was conducted using an isocratic mobile phase composed of acetonitrile and water (containing 0.1% formic acid) in a 75:25 (*v*/*v*) ratio at a flow rate of 1.0 mL/min. Chromatographic separation was achieved on an ACE C8 column (4.6 × 50 mm, 5.0 µm particle size).

Detection was carried out with an API-3000 triple quadrupole mass spectrometer (LCMS-2010A, Shimadzu, Japan) featuring an electrospray ionization (ESI) source operated in positive ion mode. The mass spectrometer settings included a desolation temperature of 700 °C, source temperature of 380 °C, ion source voltage of 5000 V, entrance potential of 10 V, and collision energy of 35 V, with additional tuning for Alectinib and the IS. Detection was achieved using multiple reaction monitoring (MRM) for specific *m*/*z* transitions.

### 2.16. Linearity, Precision, and Accuracy

The developed method was evaluated for linearity across a concentration range of 100 to 4000 ng/mL for Alectinib. Calibration curves were constructed, and the correlation coefficient (R^2^) was calculated to confirm the method’s suitability for quantitative analysis.

Precision and accuracy were determined using six replicates of Alectinib quality control (QC) samples at different concentration levels: lower limit of quantification (LLOQ), QCLow (300 ng/mL), QCMid (15,000 ng/mL), and QCHigh (34,000 ng/mL). Standard deviations and relative errors were calculated to ensure the reliability and reproducibility of the method.

### 2.17. Animal Handling and Sample Preparation

Pre-clinical pharmacokinetic investigations were conducted using six male Wistar rats (weighing 200 ± 15 g, aged 4 weeks). Prior to the experiment, the animals were fasted overnight with free access to water and were marked on the tail for individual identification. The rats were randomly divided into two groups. The control group received 20 mg/kg of free Alectinib administered orally as a suspension in distilled water. The treatment group was given an equivalent dose of Alectinib in the form of G4-NH_2_-PAMAM–Alectinib complexes, also via oral gavage. Blood samples were collected from the tail after warming for 30 s using a hot pad to facilitate sampling. A volume of 350 µL was collected at 0, 0.5, 1.5, 2, 3, 4, 6, 8, 10, 24, and 48 h, transferred into K3 EDTA tubes, centrifuged to separate plasma, and stored at −80 °C until analysis.

Sample preparation was carried out using the liquid–liquid extraction (LLE) technique. Frozen plasma samples were thawed at room temperature, vortexed to ensure uniform mixing, and combined with 500 µL acetonitrile (ACN) as diluent, 300 µL plasma, and 50 µL rosuvastatin as the internal standard. The mixture was vortexed for 2 min, centrifuged at 4000 rpm for 15 min, and 200 µL of the supernatant was transferred into LC-MS insert vials. Subsequently, 20 µL of the processed sample was analyzed using LC-MS/MS for quantification and pharmacokinetic evaluation.

### 2.18. Statistical Analysis

All data are presented as mean ± SD. Statistical analysis was performed using GraphPad Prism (version 8.0.2.236). For EE%, particle size, and zeta potential, one-way ANOVA followed by Tukey’s multiple comparisons test was used to assess significant differences among formulations using “column input”. Two-way ANOVA was used to evaluate in vitro release study time x treatment interaction followed by Šidák’s multiple comparisons (time vs. release % for each group). For the cytotoxicity and IC_50_ values, non-linear regression for dose–response curves was used and IC_50_ comparison was carried out via a sum of squares F test. Tumor volume in animals (end point evaluation) was examined using an unpaired *t*-test. *p*-values less than 0.05 were considered statistically significant. Evaluation of methods of analysis was performed according to the ICH guideline of pharmaceutical analysis.

## 3. Results

Development and Validation of the Method of Analysis of Alectinib.

### 3.1. UV-VIS Scan for λmax Determination

The determination of the maximum absorption wavelength (λmax) for Alectinib was conducted using UV-VIS spectroscopy. A sample of Alectinib, prepared at a concentration of 60 µg/mL in methanol, was scanned to identify the wavelength of maximum absorbance. The results indicated that Alectinib exhibited its highest absorbance at 340 nm. These control experiments confirmed that neither methanol nor PAMAM dendrimers interfered with the absorbance of Alectinib at the chosen λmax, thereby validating the specificity of the method.

### 3.2. HPLC-PDA Method Development and Validation

A high-performance liquid chromatography (HPLC) system equipped with a photodiode array (PDA) detector was employed for the analysis of Alectinib. The wavelength of 340 nm, previously determined as the λmax for Alectinib, was used for all measurements. A representative chromatogram of a 30 µg/mL Alectinib solution in methanol was obtained under the chromatographic conditions.

### 3.3. Linearity and Precision and Accuracy Assessment

The linearity of the HPLC-PDA method for Alectinib quantification was evaluated using six concentrations ranging from 1.875 µg/mL to 60 µg/mL. Each concentration was measured in triplicate, and the results were used to construct a calibration curve. The linear regression analysis yielded a correlation coefficient (R^2^) of 0.9997, indicating excellent linearity across the tested concentration range. As shown in Figure S1, the retention time of Alectinib was 3.37 mL/min. the method was precise and accurate and the LOD was equal to 0.763 µg/mL and LOQ was equal to 2.310 µg/mL. The LOQ is the minimum concentration that the method can quantify which means that measurement should be equal to or above this concentration to be reliable. However, this is a theoretical calculation and, in practice, it is preferable to measure within the range of linearity. Figure S2 shows the chromatogram of Alectinib.

### 3.4. Entrapment Efficiency and Drug Loading

The determination of the encapsulation efficiency (EE%) of G4-NH2-PAMAM–Alectinib dendrimer complexes was conducted and the findings are provided in Table 2. EE% refers to the conditions under which the preparation was carried out and the ease with which the drug can be entrapped in the dendrimer. The samples were prepared on a small scale, leading to a certain degree of material loss throughout the preparation process. To achieve the best possible formula and drug loading, different approaches were followed which were the addition of surfactant and stabilizer in addition to changing the ratio of drug to dendrimer.

Alectinib is a highly lipophilic drug which necessitates dissolving it in DMSO and the addition of the PAMAM to perform the entrapment. The addition of water then will result in the precipitation of the unencapsulated Alectinib and keeping the complex soluble which was the aim of the work. Evaporation of DMSO is the final step to formulate an orally safe formulation. The step of the addition of water might result in the instability of the complex, and for that reason, the stabilizer showed its effectiveness.

Using ANOVA, results in Table 2 show that EE of F1 and F2 where the amount of PAMAM was doubled gave a non-significant change in the EE% of Alectinib (*p* > 0.05).

Stabilization of NPs by PVA (F3) gave significantly higher EE than that of either F1 or F2 (*p* < 0.05). This is attributed to the stabilization effect of the PVA [24,25].

Increasing the PVA concentration in F3–F5 gave the optimum concentration used in F4 which gave EE% of 59 ± 5% which is significantly higher than that of F1–F3 (*p* < 0.05). A further increase to 15 mg resulted in a decrease in EE% as if there were no stabilizer.

PEG400 and Tween 80 also improved the EE% of Alectinib owing to their solubilization effect and decreasing the interfacial tension at the step of the addition of water. F6 gave 40 ± 5% EE and F7 53% ± 5%, which proves the importance of using surfactant when the drug is highly lipophilic.

To investigate the possibility of combining the effect of PVA and Tween 80, F8 was prepared. However, a high depletion in the EE% resulted. This might be attributed to the high solubilization power of the mixture which retained Alectinib in the organic phase and hindered its reaction with the PAMAM.

Finally, F9 was prepared to investigate the opposite design of the previous formulations where the PAMAM amount is less than Alectinib and introduced a higher concentration of PAMAM with the optimum amount of PVA. But again, the EE% decreased from 59 ± 5% (F4) to 38% ± 5% which is significantly lower (*p* < 0.05). This might be due to the crowded system, especially since both PAMAM and Alectinib are partially ionized at the pH of deionized water used (7–7.4), which might hinder the encapsulation process.

### 3.5. Characterization of G4-NH2-PAMAM–Alectinib Dendrimer

The prepared complexes were assessed to confirm the synthesis of the complexes. Subsequently, the preparation process was assessed, and the most optimal complex was selected for subsequent research. The characterization tests were performed on the selected formula (S1).

#### 3.5.1. Characterization of G4-NH2-PAMAM–Alectinib Dendrimer by FTIR Analysis

The study involved conducting Fourier transform infrared (FTIR) spectroscopy to assess and compare the chemical interactions among G4-NH_2_-PAMAM dendrimers, Alectinib, and the G4-NH_2_-PAMAM-Alectinib complex. As illustrated in Figure S3, the FTIR spectrum of pure Alectinib exhibited characteristic peaks at 2220 cm^−1^ (nitrile bond stretching), 3300 cm^−1^ (N-H stretching of secondary amide), 3053 cm^−1^ (=C-H stretching), 2954 cm^−1^ (C-H stretching), 1660 cm^−1^ (C=O stretching of ketone, slightly shifted due to conjugation effects), 1600 cm^−1^ (C=C stretching), and 1220 cm^−1^ (C-N stretching). The FTIR profile of the G4-NH_2_-PAMAM dendrimer alone showed distinct absorption bands at 3296 cm^−1^ (N-H stretching from primary amines), 2939 cm^−1^ (C-H stretching), 2829 cm^−1^ (symmetric CH_2_ stretching), and 1022 cm^−1^ (C-N stretching of tertiary amines) which is considered a fingerprint for the G4-NH2-PAMAM [26]. In the spectrum of the G4-NH_2_-PAMAM–Alectinib complex, the significant change of the 1022 cm^−1^ peak of PAMAM (almost disappearance) suggests a deep encapsulation of Alectinib [27] and the appearance of peaks at 3417 cm^−1^ (secondary amide) and 2227 cm^−1^ (nitrile) without notable shifts indicates these groups did not participate in strong interactions. However, the disappearance of peaks at 3053 cm^−1^, 2954 cm^−1^, and 1660 cm^−1^, along with the absence of dendrimer-related peaks at 3296 cm^−1^, 2829 cm^−1^, 1592 cm^−1^ and those corresponding to tertiary amines, suggests significant molecular interactions occurred between Alectinib and the dendrimer. Collectively, these spectral modifications confirm the successful formation of a G4-NH_2_-PAMAM–Alectinib complex.

#### 3.5.2. Characterization of G4-NH2-PAMAM–Alectinib Dendrimer Complex by DSC Analysis

**Figure 1:**
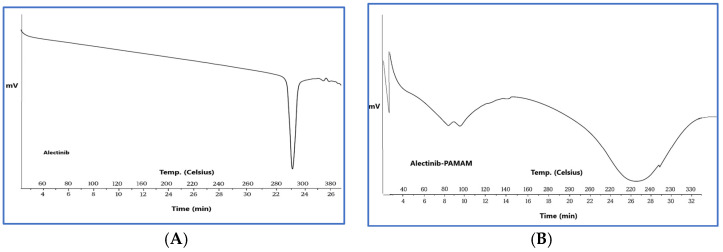
(**A**) DSC Thermogram of Alectinib showing its melting point at 270 °C, (**B**) DSC Thermogram of Alectinib-loaded G4-NH2-PAMAM (F4) complex showing a broad peak referring to the formation of the complex.

To acquire more validation, further characterization of the G4-NH2-PAMAM–Alectinib dendrimer complex was conducted using DSC. Figure 1 displays the outcomes of the Alectinib and G4-NH2-PAMAM–Alectinib dendrimer (F4) complex. To conduct a more comprehensive investigation of Alectinib hydrochloride, we analyzed its thermal behavior and observed any possible alterations in its crystallinity pattern by DSC experiments. Alectinib crystals exhibit a distinct crystalline structure, as seen by their well-defined melting point of 276.11 °C [26]. According to Figure 1, the DSC thermogram of pure Alectinib displays a distinct endothermic peak around 280–290 °C, which corresponds to its melting point and confirms its crystalline nature.

In contrast, the DSC thermogram of the Alectinib–PAMAM nanoparticle formulation exhibits a marked change in thermal behavior. Specifically, the sharp melting peak of Alectinib is either absent or significantly broadened and shifted. This change is not simply due to dilution by PAMAM but suggests a strong interaction between Alectinib and the PAMAM dendrimer, likely through hydrogen bonding or encapsulation within the dendrimer cavities. The suppression or disappearance of the drug’s melting peak is commonly observed in DSC profiles of drug–polymer complexes and is interpreted as evidence of successful encapsulation or conjugation, reduced molecular mobility of the drug due to interaction with the carrier, and possibly partial or complete amorphization.

These results support the formation of a stable Alectinib–PAMAM nanoparticle complex, which may enhance the drug’s solubility and thermal stability.

#### 3.5.3. Particle Size (PS), Polydispersity Index (PDI), and ζ-Potential of the NPs

**Figure 2:**
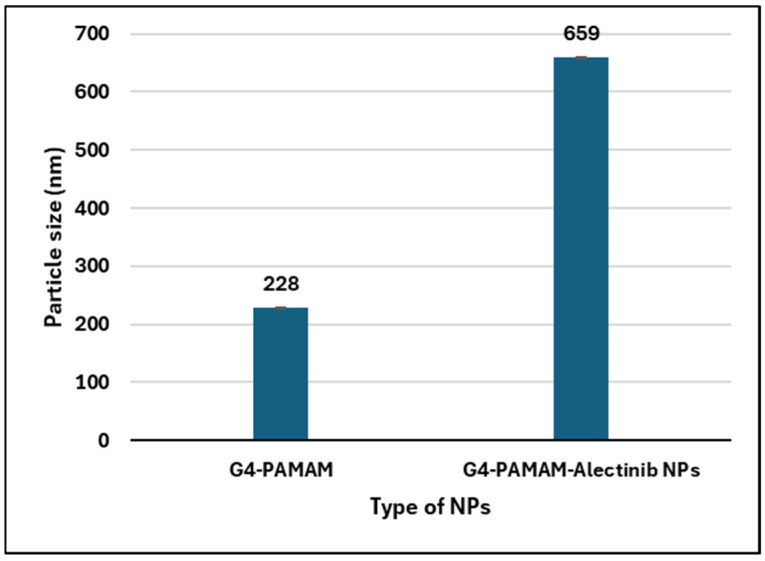
Histogram showing the particle size change in the PAMAM NPs after loading of Alectinib.

The physicochemical properties of G4-PAMAM dendrimers and G4-NH2-PAMAM–Alectinib were evaluated using a Nano Zeta-Sizer, and the result showed that the average particle size of the nanocarrier was approximately 228 nm, while the Alectinib-loaded nanocarrier had an average particle size of around 659 nm which is statistically significantly bigger (*p* < 0.05). The significant increase in size after loading the drug indicates the formation of a complex and implies the occurrence of interaction between both substances. Additionally, the polydispersity index (PDI) was found to be similar for both the nanocarrier and the new complex, indicating a monodisperse and fairly uniform distribution of nanoparticle sizes within the formulation. The complex displayed a surface charge of +16.9 mV, indicating good stability resulting from repulsive forces between particles [27]. Table 3 and Figure 2 shows the results (also, see Figures S4 and S5).

#### 3.5.4. Characterization of G4-NH2-PAMAM–Alectinib Dendrimers by SEM

**Figure 3:**
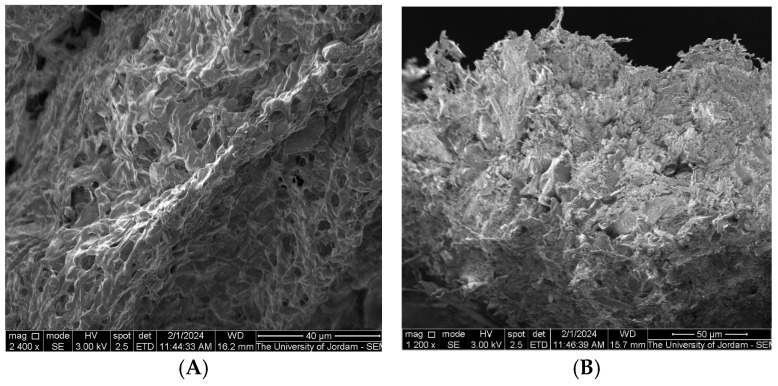
The morphology of the G4-NH2-PAMAM dendrimer nanoparticle (**A**) and G4-NH2-PAMAM Alectinib-loaded NPs (**B**).

The morphologies of the dendrimer nanoparticles were investigated with the help of SEM. The scanning electron microscopy (SEM) technique reveals that the Alectinib-loaded PAMAM showed no separated crystals of precipitated Alectinib which means that the preparation method and separation of free drug were successful, and the loaded drug was effectively encapsulated as shown in Figure 3B. Figure 3A shows the surface of unloaded PAMAM with little difference from the loaded one. These photos also proved that freeze drying did not result in separation and precipitation of the drug which might be interesting for the production of stable powder.

### 3.6. In Vitro Drug Release

The in vitro drug release test provides insight into the performance of the drug delivery system. The free drug, lacking any physical barriers, dissolves at a rate determined solely by its solubility under the test conditions. In contrast, the release rate of the encapsulated drug reflects its interaction with the nanoparticles (NPs), offering valuable information about the binding mechanism and overall system performance.

**Figure 4:**
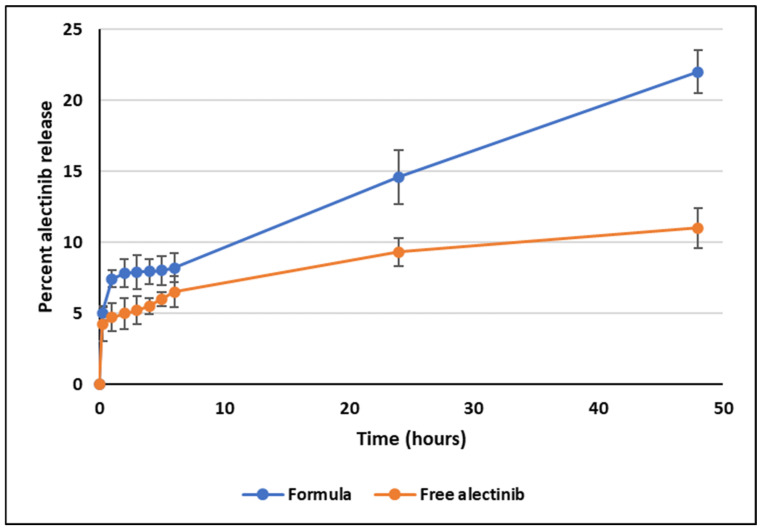
In vitro release plot of Alectinib at pH 6.8 from the dendrimer and the free drug.

The selection of the release medium and the percentage of sodium lauryl sulfate (SLS) was based on previous studies [28] to ensure sink conditions were maintained throughout the experiment. At pH 6.8, the result over 48 h was 22.2% ± 0.3, compared to 11.1% ± 0.11 for the control, representing a statistically significant increase in the total amount of Alectinib released from the prepared NPs, Figure 4. However, while this release profile suggests potential for controlled drug delivery, it remains suboptimal for immediate-release formulations, necessitating further optimization of formulation parameters.

Notably, the release pattern from 1 to 48 h demonstrated a strong correlation with zero-order kinetics (R^2^ = 0.9967), suggesting a sustained and predictable release profile. The initial release observed within the first 30 min is likely due to Alectinib adsorbed on the outer surface of the dendrimer NPs. Further studies, including extended-release experiments and evaluations of administration routes, are required to confirm the system’s suitability for controlled drug delivery applications.

The release test performed at low pH (1.2 and 2.5) did not give clear profile due to the degradation of Alectinib at low pH [28] (Figures S6 and S7).

### 3.7. Cytotoxicity Assay

**Figure 5:**
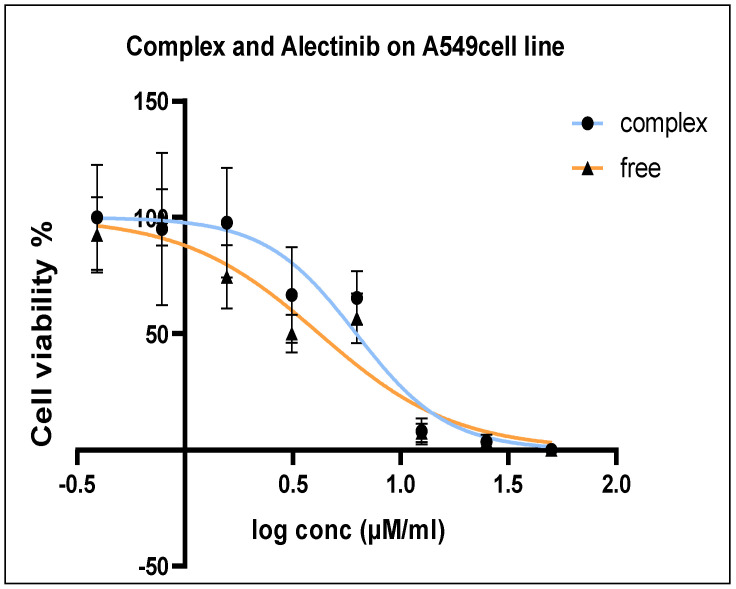
A549 Cell viability percentage after 72 h treatment in the range 100–0.781 µM/mL.

The antiproliferative effects of Alectinib and Alectinib-loaded G4-NH_2_-PAMAM on the A549 lung cancer cell line was evaluated by determining the half-maximal inhibitory concentration (IC_50_) using the MTT assay. After 72 h of treatment, the IC_50_ values for free Alectinib and Alectinib-loaded G4-NH_2_-PAMAM were 4.189 µM/mL and 6.218 µM/mL, respectively. These results indicate that both formulations exhibited comparable antiproliferative activity against A549 cells, Figure 5.

### 3.8. Clonogenic Assay

**Figure 6:**
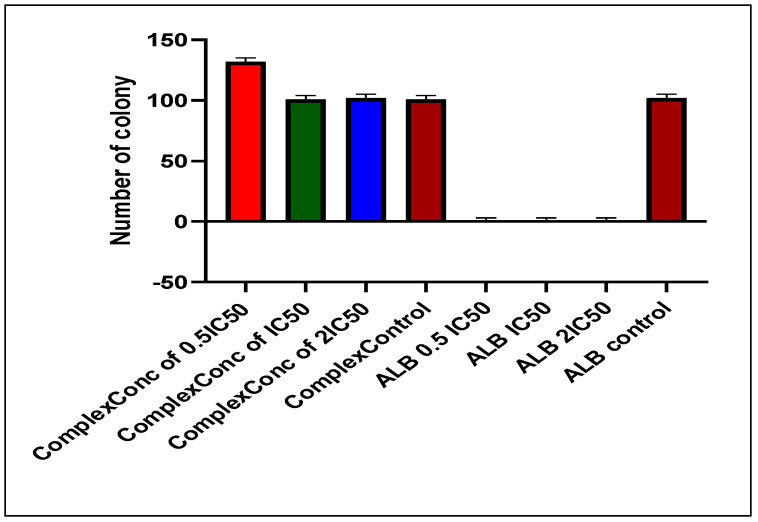
Number of colonies inhibited by Alectinib and the NPs.

The colony formation assay was conducted to assess the growth rate of A549 cells following treatment, as well as the proliferative capacity of untreated cells. After seven days of incubation post-seeding, colony formation was measured as shown in Figure 6 below.

### 3.9. Scratch-Wound Assay

**Figure 7:**
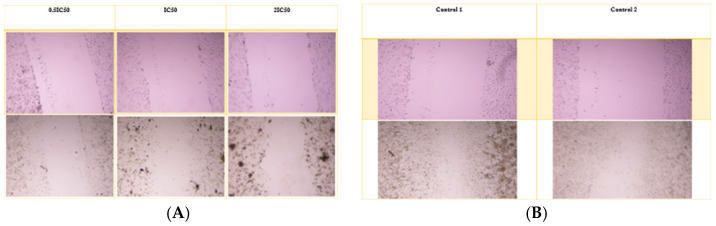
The scratch assay was conducted to evaluate the migratory potential of A549 cells following treatment with the nanoparticle complex. (**A**) The pre-treatment and post-treatment images for the complex at 0.5 IC_50_, IC_50_, and 2 IC_50_ concentrations, while (**B**) illustrates the untreated control group for comparison.

**Figure 8:**
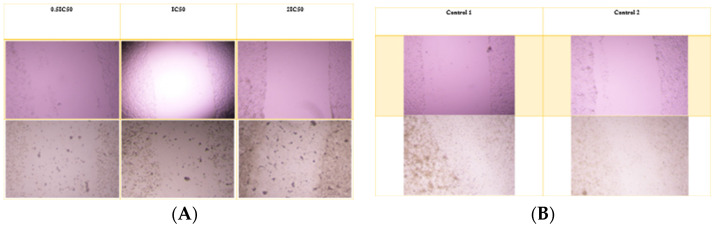
The scratch assay was performed to assess the migratory inhibition of A549 cells treated with free Alectinib. (**A**) The pre-treatment and post-treatment images for free Alectinib at 0.5 IC_50_, IC_50_, and 2 IC_50_ concentrations, while (**B**) depicts the untreated control group for comparison.

For the A549 cell line, the average wound opening percentage after 72 h was 98%, 98%, and 96% for 0.5 IC_50_, IC_50_, and 2 IC_50_ concentrations of the nanoparticle formulation, respectively. In contrast, the control group exhibited the lowest opening percentage at 17.8%. The free drug condition was not applicable for comparison, as Alectinib completely eradicated the cells. Figure 7 and Figure 8 illustrate the wound opening percentage for free Alectinib and the nanoparticle formulation, comparing the initial (day 0) and post-treatment (72 h) conditions.

### 3.10. In Vivo Xenograft Assay

To establish an immunosuppressed xenograft model, dexamethasone (5 mg/kg) was administered to all mice for 21 days to suppress their immune response (Figure S8). Following immunosuppression, 2.5 × 10^6^ H460 large lung cancer cells were subcutaneously injected into the mice, and tumor growth was monitored every day. This approach was optimized as an efficient methodology for inducing human tumors in BALB/c mice, specifically designed for pre-clinical investigations [29].

After 14 days, tumor size was measured using a caliper, revealing an average tumor diameter of approximately 4 mm. Throughout the study, the mice were closely monitored for behavioral changes and regularly weighed. The results indicated that the mice maintained a stable weight and exhibited no signs of systemic toxicity, confirming the administration of a non-harmful yet potent dosage, as previously determined by [30].

**Figure 9:**
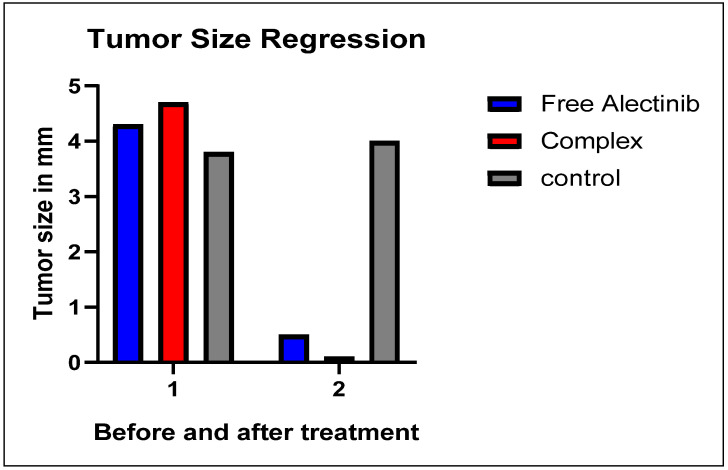
Regression of Tumor Size After 17 Days of Treatment.

Treatment was conducted over 17 days following ethical guidelines, after which the mice were euthanized using isoflurane inhalation. Upon examination of the tumor sites, the group treated with free Alectinib exhibited a minimal amount of residual tumor tissue. In contrast, the group treated with G4-NH_2_-PAMAM–Alectinib showed a complete disappearance of the tumor. Meanwhile, the control group displayed continuous tumor growth throughout the 17-day period, with no significant reduction in tumor size (Figure 9).

### 3.11. LC/MS/MS Method of Analysis of Alectinib in Rat Plasma

**Figure 10:**
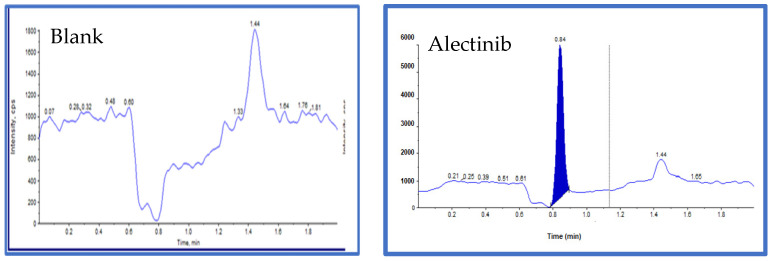
The blank sample with no peak of Alectinib and Alectinib (standard) 483.300 Da, height: 5.66 × 10^3^ cps, RT: 0.843.

The developed analytical method was employed to quantify Alectinib in rat plasma samples. Figure 10 presents the chromatogram of blank plasma and a representative chromatogram of Alectinib. The detected ion exhibited a molecular weight of 483.3 Da, with a retention time (RT) of 0.843 min. These results demonstrate that the method is rapid, cost-effective, and efficient, making it well-suited for high-throughput pharmacokinetic analysis.

The established analytical method was validated by following FDA guidelines for bioanalytical method validation in the United States. The sensitivity, stability, linearity, and selectivity were measured to ensure method validation. The linearity was established for the range of 10.0–4000.0 ng/mL of Alectinib in human plasma.

#### 3.11.1. Linearity and Precision and Accuracy Assessment

The developed method was evaluated for linearity over a concentration range of 100 to 4000 ng/mL for Alectinib. Calibration curves were generated, and the correlation coefficient (R^2^) was determined to assess the method’s suitability for quantitative analysis (Figure S9).

Precision and accuracy were assessed using six replicates of Alectinib quality control (QC) samples at different concentration levels: lower limit of quantification (LLOQ), QCLow (300 ng/mL), QCMid (15,000 ng/mL), and QCHigh (34,000 ng/mL). Standard deviations and relative errors were calculated to ensure the reliability and reproducibility of the method (Table S1).

#### 3.11.2. Pharmacokinetic Study

**Figure 11:**
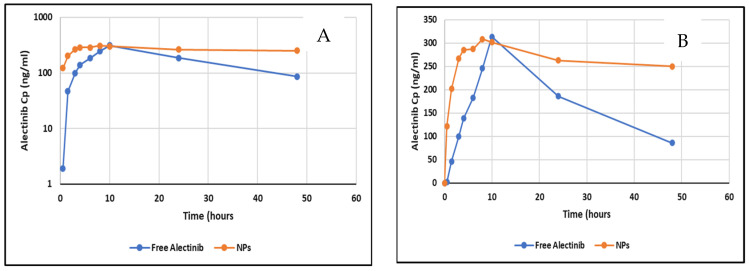
(**A**) Log transformed plot of Cp vs. profile of free Alectinib and NPs. (**B**) Plasma level–time profile of free Alectinib and Alectinib–PAMAM NPs.

Plasma level–time profiles of Alectinib from the NPs and the free Alectinib are illustrated in Figure 11.

The pharmacokinetic analysis revealed that the nanoparticle (NP) formulation exhibited a C_max_ that was not significantly different from that of free Alectinib. However, the T_max_ was significantly reduced, indicating a faster absorption rate. The absorption rate constant (K_a_), calculated under the assumption of linear absorption, was 0.43 h^−1^ for free Alectinib and 0.68 h^−1^ for Alectinib formulated in NPs, further supporting the enhanced absorption of Alectinib from the nanoparticle system. This improvement may be attributed to the enhanced dissolution and permeability properties conferred by the NP formulation.

The AUC_0–t_ was significantly higher when using PAMAM NPs as a drug delivery system for oral administration of Alectinib. The extent of bioavailability increased by 36% compared to free Alectinib, as measured by the AUC parameter, which quantifies drug absorption. A similar trend was observed when using AUC_0–∞_, though the extrapolated area of the NP formulation exceeded 20% of the total area, limiting direct comparison to AUC_0–t_. Table 4 shows the basic PKP of free Alectinib versus Alectinib-loaded PAMAM NPs.

Furthermore, the prolonged elimination half-life of Alectinib in the NP formulation suggests an extended residence time in the body. This could be attributed to the sustained release of Alectinib from the absorbed NPs, which may contribute to prolonged systemic exposure and enhanced therapeutic potential.

## 4. Discussion

NSCLC is a type of cancer which does not often respond to therapy. This may be associated with different kinds of mutations in genes such as anaplastic lymphoma kinase (ALK), proto-oncogene B-Raf, discoidin domain receptor tyrosinek2 (DDR2), and EGFR.

The results of this study demonstrate the potential of G4-NH2-PAMAM dendrimers as a nanocarrier system for improving the pharmacokinetics and therapeutic performance of Alectinib in NSCLC. Alectinib, while clinically effective against ALK-positive NSCLC, is hindered by its low water solubility and limited bioavailability. By forming dendrimer NPs, we addressed these challenges and achieved promising enhancement in formation stability, release behavior, cellular efficacy, and in therapeutic efficacy.

The optimal formulation (F4) achieved an encapsulation efficiency (EE%) of 59 ± 5% facilitated by PVA. This significantly surpassed the EE values of surfactant-free formulations (F1 and F2), underscoring the importance of stabilizers in enhancing drug entrapment, particularly for hydrophobic agents like Alectinib.

Formulations are evaluated in Table 2. EE% depends on formulation conditions and drug entrapment efficiency within the dendrimer. Due to the small-scale preparation, some material loss occurred. Various strategies, including surfactant/stabilizer addition and drug-to-dendrimer ratio adjustments, were explored to optimize drug loading, and ANOVA results showed no significant EE% change when doubling PAMAM (F1, F2; *p* > 0.05). Stabilization with PVA (F3) significantly increased EE% (*p* < 0.05), with the optimal PVA concentration in F4 achieving 59 ± 5% EE, significantly higher than that of F1–F3 (*p* < 0.05). Excessive PVA (F5) or increased PAMAM (F9) reduced EE%, likely due to system overcrowding and ionization effects at pH 7–7.4, hindering encapsulation [31].

The loading efficiency of Alectinib onto the PAMAM was aligned with EE% of the drug. Since the amount of the drug is fixed in all formulations except F2 and F9, the higher the EE%, the higher the loading capacity was. F2 and F9 showed the least loading capacity due to the higher amount of PAMAM used which was concluded not to have an improvement effect on the encapsulation of Alectinib.

The interpretation of FTIR spectra of Alectinib hydrochloride can be summarized by the disappearance of the fingerprint peak of PAMAM at 1022 cm^−1^, shortening of 2220 cm^−1^ (fingerprint of nitrile bond stretching), and disappearance of peaks at 3053 cm^−1^, 2954 cm^−1^, and 1660 cm^−1^, strongly suggesting an interaction between PAMAM and Alectinib.

DSC analysis showed a broad peak instead of the sharp melting peak of Alectinib, confirming the entrapment of Alectinib in the PAMAM.

At pH 6.8, Alectinib-loaded NPs achieved a total drug release of 22.2% ± 0.3, over 48 h, significantly exceeding the 11.1% ± 0.11 observed for free Alectinib. Although this increase is statistically significant, the release remains insufficient for immediate-release formulations, necessitating further optimization. However, the sustained-release profile followed zero-order kinetics from 1 h to 48 h (R^2^ = 0.9967), indicating potential for controlled-release applications. The initial release within the first 30 min likely resulted from surface-bound Alectinib, while extended studies are required to refine release kinetics and optimize the administration route.

Comparable release behavior has been reported with other delivery systems. For example, chitosan–alginate NPs loaded with Alectinib showed enhanced release in simulated gastrointestinal fluids [32] while lipid-based nanocarriers like SNEDDS demonstrated rapid dissolution but lack sustained control [9]. In contrast, dendrimers offer a customizable architecture allowing fine tuning of release kinetics and drug–dendrimer interaction.

Alectinib demonstrated potent antitumor activity in both in vitro and in vivo models against tumor cell lines harboring ALK gene alterations, including NSCLC cells with the EML4-ALK fusion. Moreover, it was reported that dendrimers—owing to their defined structure, high safety, low immunogenicity, capacity to cross biological barriers, prolonged circulation stability, and targeted delivery ability—have been extensively employed for gene and drug delivery applications [33].

Several published studies showed the IC_50_ of Alectinib on different types of cells. Song et al., 2020 studied three types of cells; on KARPAS 299, Alectinib IC_50_ was 3 nM, that for nB1 (neuroblastoma) was 4.5 nM, and that for NCI-H2228 (lung cancer) was equal to 53 nM [34].

In another study in a drug resistance context, Delmonte et al. used resistance-induced overexpressed TGF-α HGF cells and the IC_50_ of Alectinib was measured as 38.2 nM [35].

In multi-drudge resistance studies, the IC_50_ of Alectinib exceeded 10 µM [36].

The reason behind our results of micromolar levels is that A549 cells lack the ALK fusion oncogene which shows the highest sensitivity to Alectinib and they express p-Gp efflux (KRAS) that contributes to the decreased sensitivity to Alectinib. On A549 cells, Alectinib mainly shows “off-target kinase inhibition” besides modulation of ABCB1 and ABCG2 efflux transporters. Ata et al. also calculated IC_50_ of Alectinib-loaded chitosan–alginate nanoparticles as 3.29 μg/mL compared to 9.63 μg/mL for the free drug.

The aim here is to test the efficacy of the PAMAM NPs in comparison to the free drug to ensure that the drug is able to release from the NPs and exert its expected effect on targets. The decrease in the IC50 might be attributed to the controlled release that keeps the drug in contact with the cells without rapid clearance.

Other nanocarriers like liposomes, polymeric micelles, and niosomes have been explored for TKI delivery. Fatani et al. reported dendrimer-encapsulated Erlotinib enhanced cytotoxicity and intracellular uptake in resistant lung cancer lines [15]. Compared to such systems, PAMAM dendrimers offer higher surface functionality, enabling both passive and active targeting modification.

The xenograft study demonstrated that the Alectinib–dendrimer complex achieved complete tumor regression in H460-bearing mice after 17 days, significantly outperforming free Alectinib (75% reduction). Importantly, no systemic toxicity was observed, supporting the biocompatibility of the formulation.

The improvement of bioavailability of orally administered Alectinib as a drug delivery system was reported by Majeed et al. Alectinib was used with cyclodextrin and the bioavailability was improved by 50%, showing an increase in Cmax and AUC_0–t_ [13]. Chitosan–alginate loaded NPs also increased the bioavailability of Alectinib by 78% [21].

In this study, pharmacokinetic analysis further confirmed enhanced absorption (lower Tmax), prolonged half-life (36.6 vs. 15.4 h), and increased extent of bioavailability by increasing AUC_0–t_ (13,456 vs. 9842 ng.h/mL), validating sustained release and protection from premature degradation in vivo. This aligns with previous studies on PAMAM-based carriers for anticancer agents which have shown increased circulation time and improved tissue targeting. The performance observed here compares favorably to a Alectinib-loaded cyclodextrin complex, which improved solubility but lacked the same sustained systemic exposure.

## 5. Conclusions

This study successfully developed a G4-NH2-PAMAM-dendrimer-based delivery system for Alectinib, demonstrating significant improvement in both therapeutic efficacy and pharmacokinetic profile. A validated and reliable HPLC method was established for the quantification of Alectinib. The drug was efficiently encapsulated within G4-NH2-PAMAM dendrimers, achieving an encapsulation efficiency of 59 ± 5%, as confirmed by FTIR and DSC. In vitro release studies at pH 6.8 showed a sustained drug release profile over 48 h. Cytotoxicity assays demonstrated a time-dependent antiproliferative effect, with 98% inhibition of cell migration at lower concentrations. In vivo studies in xenografted mice showed complete tumor regression with the nanoparticle formulation, outperforming free Alectinib. Furthermore, a cost-effective LC-MS/MS method was developed for plasma quantification, facilitating pharmacokinetic studies. Results indicated improved absorption, bioavailability, and half-life, supporting sustained drug release and reduced toxicity. These findings highlight the potential of G4-NH2-PAMAM–Alectinib nanoparticles for enhancing cancer treatment efficacy and safety.

## Figures and Tables

**Table 1:** Composition of the prepared formulations.

Formulation Code	Amount of Alectinib *w*/*w*	Amount of PAMAM (Solution Contains 1 g in 10 mL Methanol) *w*/*v*	Amount of Surfactant/Stabilizer *w*/*v*
F1	10 mg	20 µL (2 mg)	-
F2	10 mg	40 µL (4 mg)	-
F3	10 mg	20 µL (2 mg)	5 mg of PVA
F4	10 mg	20 µL (2 mg)	10 mg PVA
F5	10 mg	20 µL (2 mg)	15 mg PVA
F6	10 mg	20 µL (2 mg)	2 drop of PEG 400
F7	10 mg	20 µL (2 mg)	2 drop of Tween 80
F8	10 mg	20 µL (2 mg)	10 mg PVA + 2 drops Tween 80
F9	10 mg	250 µL (25 mg)	10 mg PVA

**Table 2:** Encapsulation Efficiency (EE%) and drug loading of G4-NH2-PAMAM–Alectinib Dendrimer complex.

Formulation Code	Amount of Alectinib *w*/*w*	Amount of PAMAM (Solution Contains 1 g in 10 mL Methanol) *w*/*v*	Amount of Surfactant/Stabilizer *w*/*v*	EE%	Drug Loading (%)
F1	10 mg	20 µL (2 mg)	-	13 ± 2%	39.3 ± 1.5%
F2	10 mg	40 µL (4 mg)	-	11% ± 2%	21.5 ± 2%
F3	10 mg	20 µL (2 mg)	5 mg of PVA	27 ± 5%	57.4 ± 3%
F4	10 mg	20 µL (2 mg)	10 mg PVA	59 ± 2%	74.6 ± 2.5%
F5	10 mg	20 µL (2 mg)	15 mg PVA	10 ± 2%	33.3 ± 2.0%
F6	10 mg	20 µL (2 mg)	2 drops of PEG 400	40 ± 2%	66.6 ± 4.1%
F7	10 mg	20 µL (2 mg)	2 drops of Tween 80	53% ± 2%	72.6 ± 4.5%
F8	10 mg	20 µL (2 mg)	10 mg PVA + 2 drops Tween 80	10% ± 2%	33.3 ± 2.2%
F9	10 mg	250 µL (25 mg)	10 mg PVA	38% ± 5%	13.1 ± 1.5%

**Table 3:** Measurement of the physicochemical properties of PAMAM and G4-NH2-PAMAM Dendrimer (F4).

Nanoparticles Composition	Particle Size ± SD	Zeta Potential ± SD	PDI ± SD
G4-PAMAM dendrimer	228 nm ± 20	10.5 m.v ± 1	0.3 ± 0.01
G4-NH2-PAMAM–Alectinib dendrimer (F4)	659 nm ± 25	16.9 m.v ± 3	0.26 ± 0.02

**Table 4:** Basic PKP parameters of free Alectinib and loaded NPs.

Parameter	Free Alectinib	Alectinib G4-NH2-PAMAM Nanoparticles
C-max (ng/mL)	313.83 ± 150.83	315.51 ± 163.14
T-max (h)	9.33 ± 1.1	* 6 ± 2
AUC0-t (ng·h/mL)	9842.03 ± 3986	* 13,455.74 ± 2756.4
AUC0-inf (ng·h/mL)	11,758.84 ± 4397.42	27,379.72 ± 7931.7
Kel (h^−1^)	0.045 ± 0.01	* 0.0189 ± 0.009
T_1/2_ (hrs)	15.4 ± 1.6	* 36.6 ± 2.5

* Indicates statistically significant differences (*p* < 0.05).

## Data Availability

All data is included in this manuscript and available according to the rules of the journal.

## Supplementary Materials

The following supporting information can be downloaded at: https://www.mdpi.com/article/10.3390/pharmaceutics17080974/s1.
