# Supplementary material for: In Vitro and In Vivo Evaluation of Alectinib-Loaded Dendrimer Nanoparticles as a Drug Delivery System for Non-Small Cell Lung Carcinoma
Source: Pharmaceutics. 2025 Jul 28;17(8):974. doi: 10.3390/pharmaceutics17080974 (PMC12389006; doi:10.3390/pharmaceutics17080974)
Supplement: Supplementary file 1 [file pharmaceutics-17-00974-s001.zip › pharmaceutics-3626036-supplementary.pdf]

Supplementary data

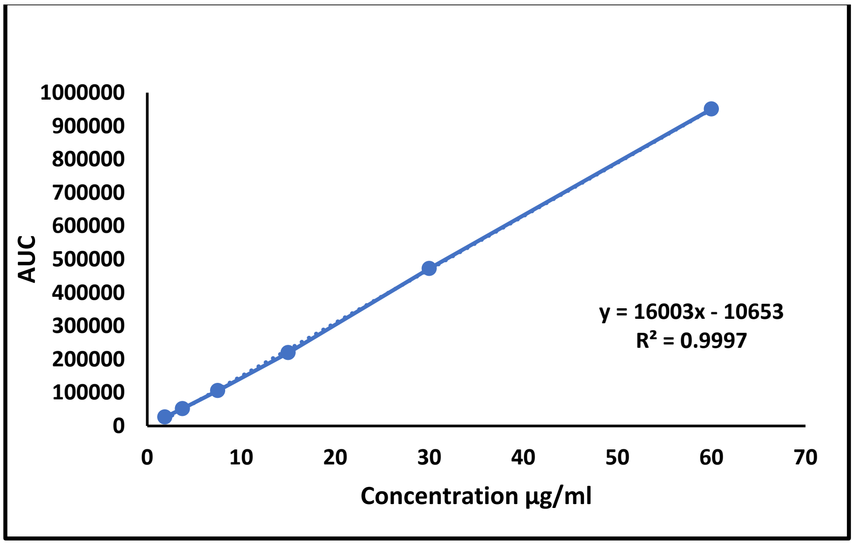

Figure S1. Linearity and a calibration curve of Alectinib show a correlation coefficient of 0.9997.

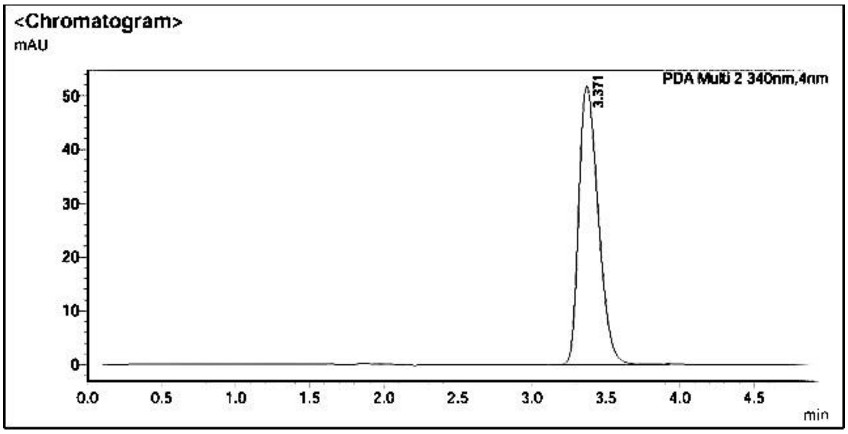

Figure S2. A chromatogram of Alectinib (15µg/ml) showing the peak and the retention time of 3.371 min.

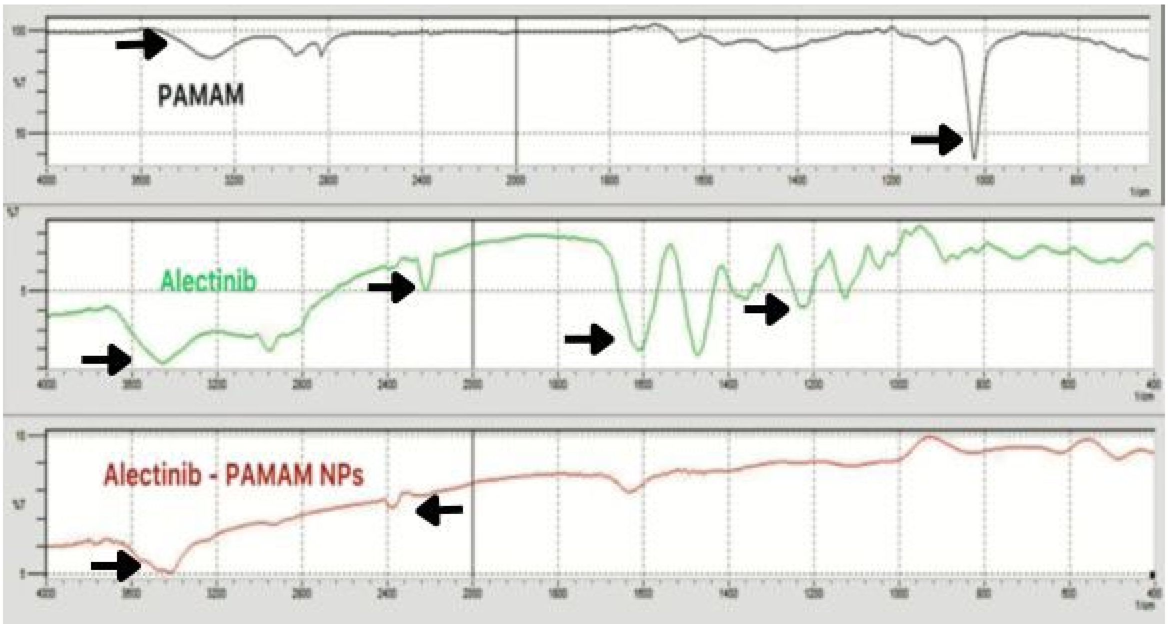

Figure S3. FTIR profile of Alectinib ,G4NH2 PAMAM dendrimer alone. and G4-NH2 PAMAM dendrimer loaded Alectinib.

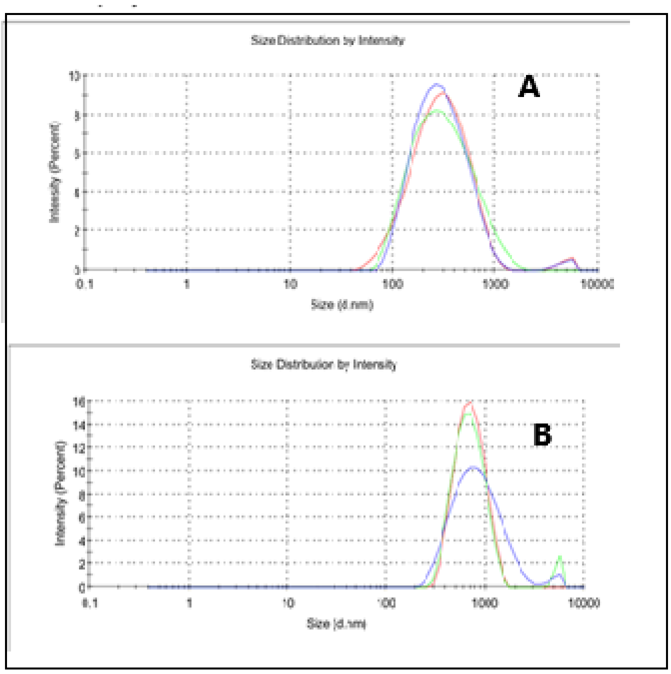

Figure S4. The particle size of G4-NH4-PA MAM dendrimers (A), and G4-NH4-PAMAM -Alectinib dendrimer NPs (B).

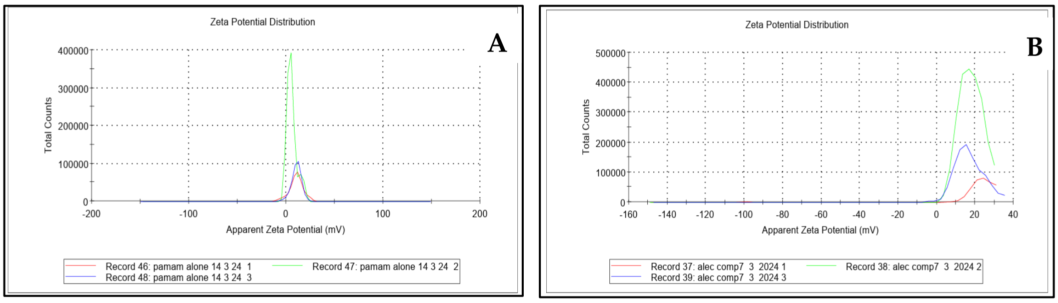

Figure S5. Zeta charge of G4-NH2-PAMAM dendrimers (A), and of G4-NH4-PAMAM -Alectinib dendrimer NPs (B).

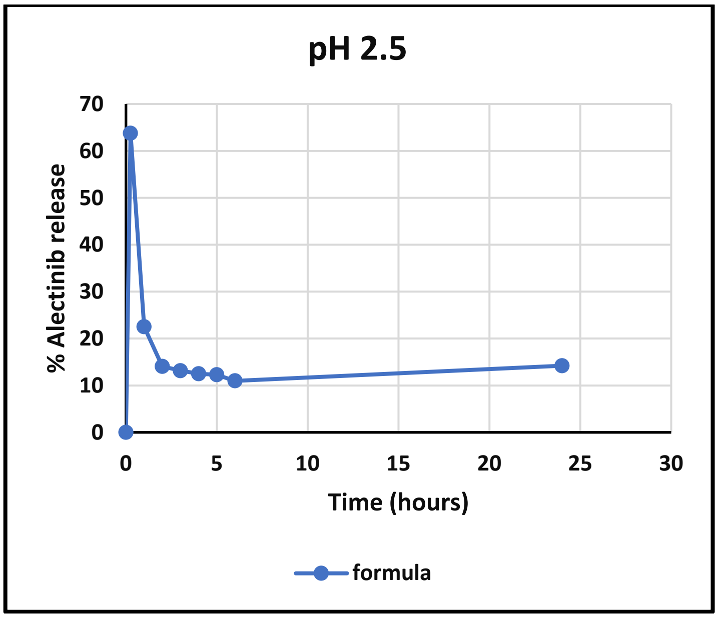

Figure S6. Release profile of PAMAM-Alectinib NPs showing a drop in the concentration of drug with time due to degradation.

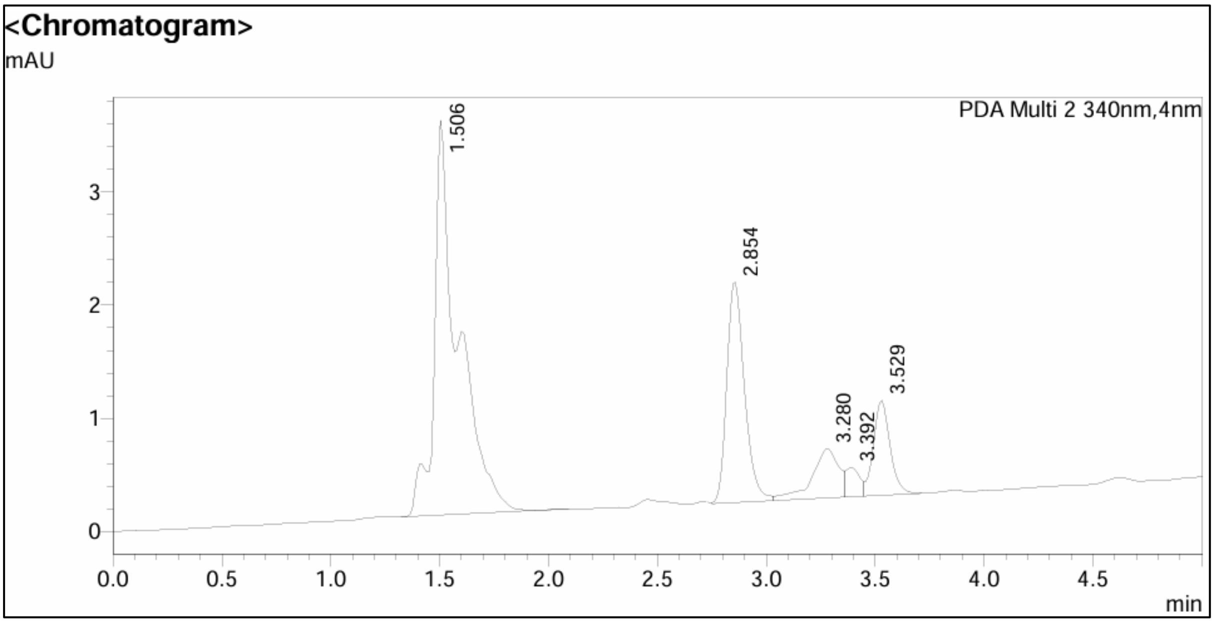

Figure S7. Chromatogram of the release of Alectinib at pH 1.2 and 2.5 that shows the degradation of Alectinib.

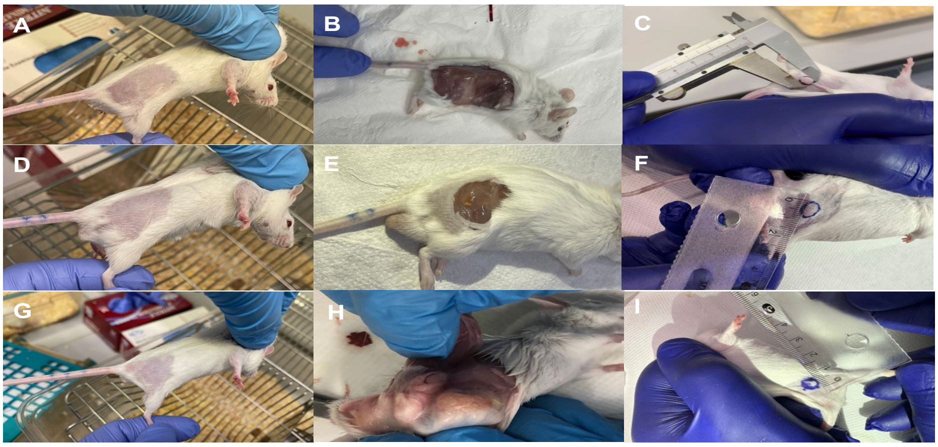

Figure S8. A schematic represented three groups of pretreatments and after treatment (A, B, C treated with complex), (D, E, F treated with free Alectinib), and (G, H, I negative control). Note: the right column represents day 0 while the other two columns represent day 17 of treatment.

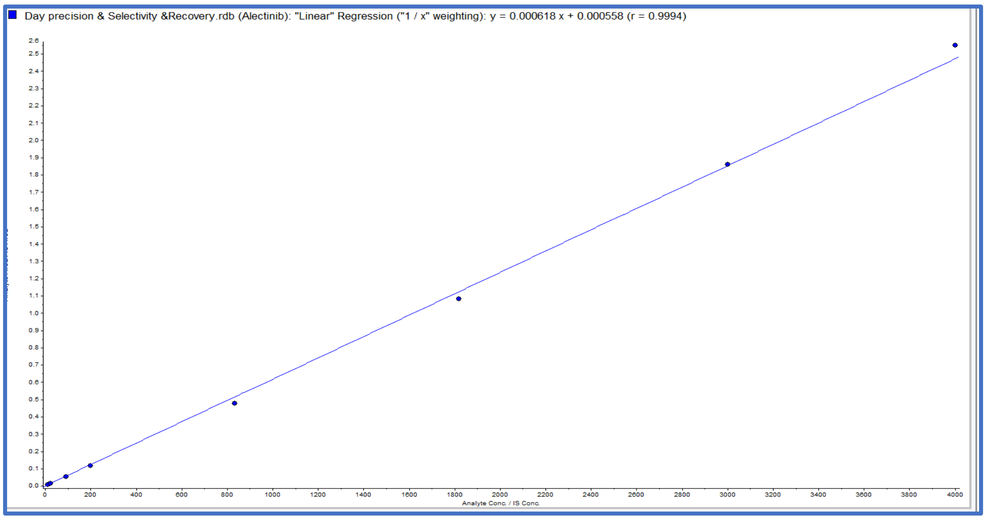

Figure S9. Calibration curve of Alectinib in rat's plasma

Table S1. Results of precision and accuracy of Alectinib in rats' plasma.

Sample Code	LLOQ (10 ng/ml)	QC Low (30 ng/ml)	QC Medium (1500 ng/ml)	QC High (3500 ng/ml)
**Replicate 1**	10.35	31.81	1616.24	3641.05
**Replicate 2**	9.72	31.93	1570.56	3720.46
**Replicate 3**	10.73	30.58	1628.85	3532.84
**Replicate 4**	9.53	29.32	1580.24	3455.65
**Replicate 5**	11.01	28.56	1610.56	3521.24
**Replicate 6**	10.98	31.25	1598.33	3487.51
**Average**	10.39	30.58	1600.80	3559.79
**STDV**	0.64	1.37	22.20	100.70
**CV%**	6.15	4.50	1.39	2.83
**Accuracy %**	103.00	101.93	106.72	101.71
